# An Unlikely Aid: Fighting HIV through TV

**Published:** 2017-12

**Authors:** Hamed EBRAHIMZADEH LEYLABADLO, Shirin SHAHSAVARI, Amir MOHAMMADZADEH, Hossein BANNAZADEH BAGHI

**Affiliations:** 1. Students’ Research Committee, Tabriz University of Medical Sciences, Tabriz, Iran; 2. Immunology Research Center, Tabriz University of Medical Sciences, Tabriz, Iran; 3. Dept. of Biotechnology, Faculty of Biological Sciences, Alzahra University, Tehran, Iran; 4. Drug Applied Research Center, Tabriz University of Medical Sciences, Tabriz, Iran; 5. Infectious and Tropical Diseases Research Center, Tabriz University of Medical Sciences, Tabriz, Iran; 6. Dept. of Microbiology and Virology, Faculty of Medicine, Tabriz University of Medical Sciences, Tabriz, Iran

## Dear Editor-in-Chief

Prevention is a major factor in fighting HIV epidemics in the world. However, a main obstacle for prevention is the lack of knowledge on HIV and its transmission ways, so reinventing ways to spread more awareness about HIV/AIDS is of utmost importance. A novel way to do this is by making use of mass media in order to influence policies and public opinions, to create a supportive environment and finally to enable the prevention of the disease. TV is the main source of information for the general Iranian population, although other sources such as radio and the internet are also used frequently. Unfortunately, information on HIV transmission methods was largely overlooked previously, due to taboos, which resulted in a drastic increase of AIDS infection statistics in Iran (1, 2). Broadcasters in general, carry a large responsibility in the Iranian society and should use this elevated position to educate and to raise awareness of serious issues such as HIV/AIDS. Most Iranian youngsters remain uneducated on HIV/AIDS and sexually risky behavior. Head of the Center for HIV/AIDS Research in Iran blames the media for not taking a stance in the health issue. The valuable information could be put out there instead of some advertisement during popular Iranian broadcasts such as soccer games, where commercial pop-ups are the norm (3).

However recently, a TV series called” Paria” aired on national media and has effectively changed the taboo surrounding HIV/AIDS. This, coupled with the fact that national media has not paid attention to this disease until now, has made, “Paria” a bold and controversial TV series with a much greater impact on the audience than dialogue-driven programs (4). In fact, referral to “HIV/AIDS” counseling and testing in Iran after airing this show has greatly increased (5).

This seems to be a positive step for Iran towards achieving the WHO`s goal to eliminate AIDS-related deaths by 2030 around the globe. However, this is only the beginning for Iran and in order to target seriously this goal the Iranian Deputy Minister of Health and the national media broadcaster IRIB must increase cooperation for decreasing infections and finally eliminating “AIDS”. Iran still has a long way to go, but incorporating media in the fight against HIV/AIDS seems to be an unlikely, yet effective, way by which to achieve this.
